# Supplementary material for: Glycemic Index and Insulinemic Index of Foods: An Interlaboratory Study Using the ISO 2010 Method
Source: Nutrients. 2019 Sep 13;11(9):2218. doi: 10.3390/nu11092218 (PMC6770275; doi:10.3390/nu11092218)
Supplement: Supplementary file 1 [file nutrients-11-02218-s001.pdf]

Table S1: Maximum concentrations (Cmax) for glucose and insulin.

Food	Lab 1 (n=15)	Lab 2 (n=15)	Lab 3 (n=13)	Overall (n=43)
		Glucose Cmax (mmol/L)		
RM Biscuit	7.5±0.7	7.2±0.6	7.3±0.86	7.35±0.11[c]
SRM Biscuit	7.6±1.1	7.5±0.7	7.7±0.81	7.61±0.14[cd]
Cracker	7.40±1.1	7.3±0.8	7.6±0.57	7.42±0.13[c]
White bread	8.2±0.9	7.8±0.7	8.1±1.18	7.99±0.15[bd]
Corn flakes	8.4±1.3	8.4±0.7	8.4±1.3	8.40±0.17[b]
Ginger-bread	9.1±1.0	9.2±0.8	9.4±0.75	9.22±0.13[a]
Glucose	9.3±0.7	9.2±0.9	9.2±0.92	9.20±0.13[a]
				0.6490*
		Insulin Cmax (mIU/L)		
RM Biscuit	168±66	258±84	222±174	217±18[bc]
SRM Biscuit	174±78	282±150	204±78	218±18[bc]
Cracker	186±72	246±186	246±126	227±21[bc]
White bread	192±66	294±120	216±66	233±15[bc]
Corn flakes	180±78	246±126	186±60	205±15[b]
Ginger-bread	186±78	264±102	252±114	237±15[c]
Glucose	234±78	348±156	264±84	271±19[a]

Values are means±SD. RM=rotary molded; SRM=sandwiched rotary molded.
[abcd] Means not sharing the same letter superscript differ significantly (Tukey's $p<0.05$).
* Significance of test-meal×laboratory interaction.

Table S2: Peak rise for glucose and insulin.

Food	Lab 1 (n=15)	Lab 2 (n=15)	Lab 3 (n=13)	Overall (n=43)
		Glucose peak rise (mmol/L)		
RM Biscuit	2.3±0.7	2.3±0.5	2.3±0.9	2.29±0.10[d]
SRM Biscuit	2.4±1.1	2.5±0.7	2.7±0.8	2.55±0.13[cd]
Cracker	2.2±1.1	2.3±0.6	2.5±0.7	2.34±0.12[d]
White bread	3.0±0.9	2.7±0.6	3.0±1.1	2.89±0.14[bc]
Corn flakes	3.1±1.2	3.4±0.6	3.3±1.3	3.26±0.16[b]
Ginger-bread	3.9±1.0	4.2±0.7	4.4±0.8	4.14±0.13[a]
Glucose	4.1±0.7	4.1±0.8	4.1±0.9	4.10±0.12[a]
				0.6096*
		Insulin peak rise (pmol/L)		
RM Biscuit	147±62	231±83	195±168	191±17[b]
SRM Biscuit	152±76	250±144	188±77	192±17[b]
Cracker	163±72	221±179	215±122	200±20[b]
White bread	161±66	263±115	187±61	206±14[b]
Corn flakes	154±77	219±123	158±51	177±14[b]
Ginger-bread	162±77	239±97	226±103	211±15[b]
Glucose	207±76	320±154	230±78	244±19[a]
	147±62	231±83	195±168	0.6430*

Values are means±SD. RM=rotary molded; SRM=sandwiched rotary molded.
[abcd] Means not sharing the same letter superscript differ significantly (Tukey's $p<0.05$).
* Significance of test-meal×laboratory interaction.

Figure S1: Intra- and Inter-laboratory variation of GI and II.

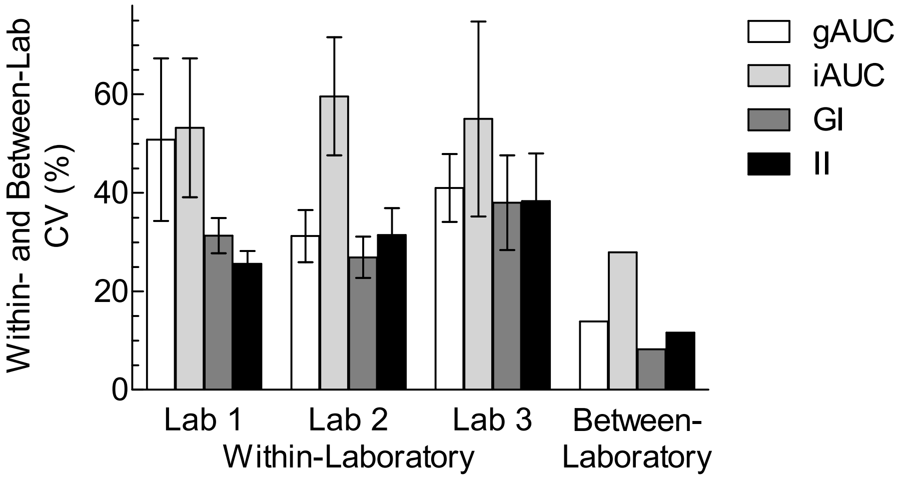

Within-laboratory variation; values are means±SD of the coefficients of variation (CV=100×SD/mean) of the gAUC (glucose iAUC), iAUC (insulin AUC), GI and II values of the 6 test-foods within each lab. Between-laboratory variation: values are the mean of the CV of the 3 lab mean values for each food. Data are from Tables 3 and 4.

Figure S2: Glucose and insulin response curves.

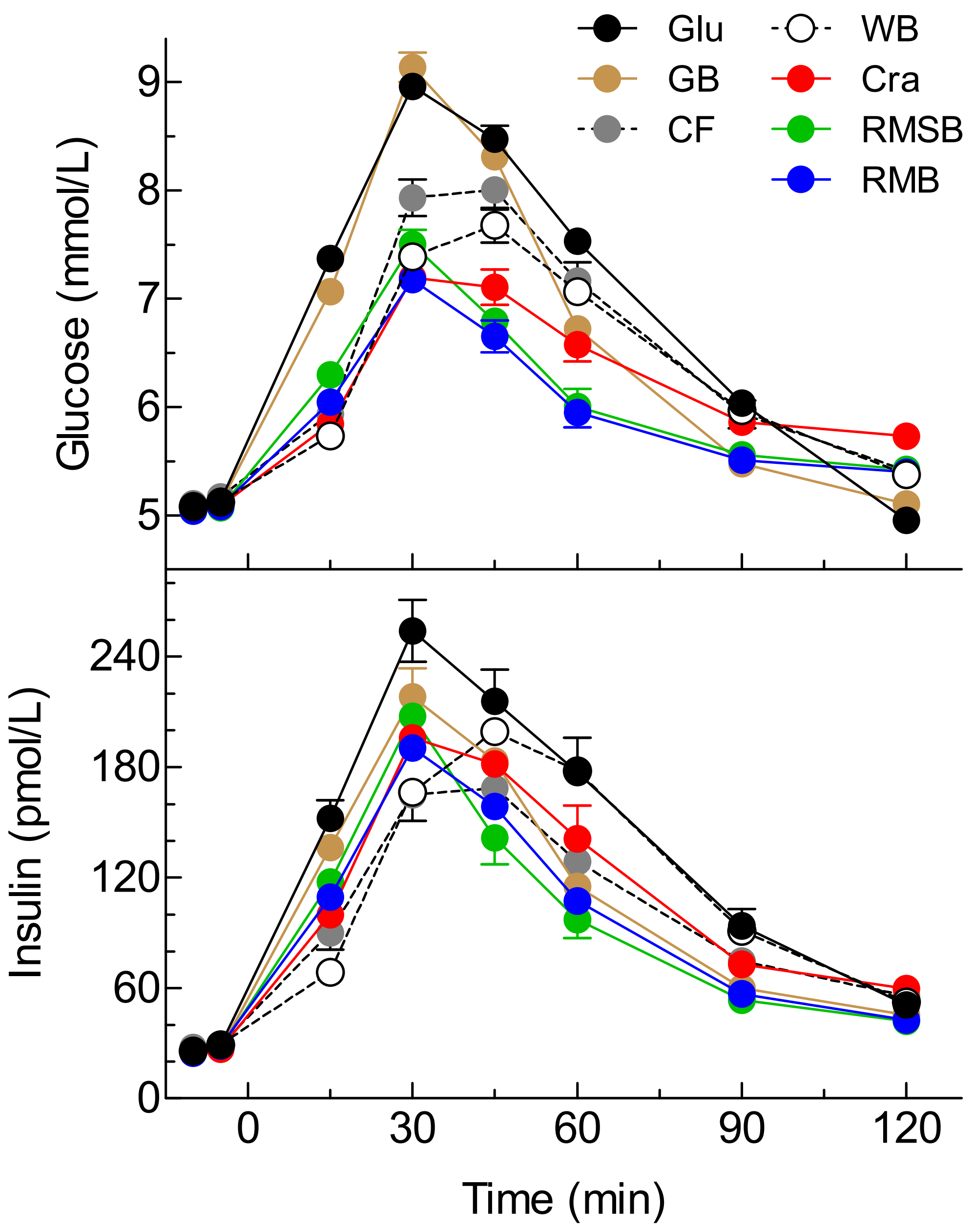

Values are means±SEM for n=43 participants. Error bars are not shown if they are smaller than the symbol or overlap other symbols or error bars. Glu, glucose; GB, ginger bread; CF, corn flakes; WB, white bread; Cra, cracker; RMB rotary molded biscuit; SRMB sandwiched rotary molded biscuit.

Figure S3: Relationship between glycemic- and insulinemic-indices before (panel A) and after (panel B) adjusting glycemic index for the protein and fat contents of the test-foods.

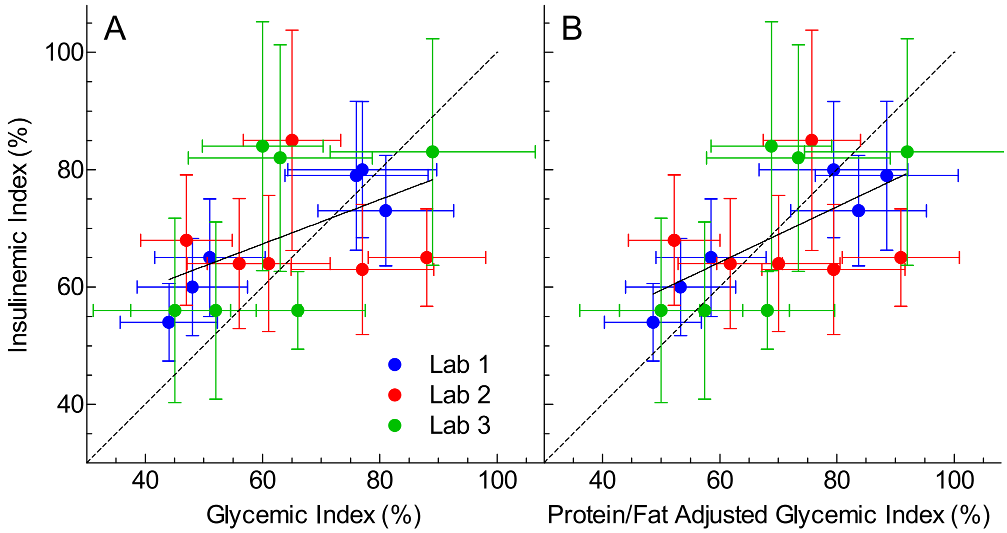

Values are means±95% confidence intervals. Dashed lines are the lines of identity; solid lines are the regression lines: panel A, r=0.512, p=0.030; panel B, r=0.623, p=0.006.
